# Catheter Ablation and Device Therapy in Patients With Transthyretin Amyloid Cardiomyopathy: A Review of Non‐Pharmacological Therapy

**DOI:** 10.1002/joa3.70281

**Published:** 2026-02-06

**Authors:** Hisanori Kanazawa, Tadashi Hoshiyama, Shozo Kaneko, Yusei Kawahara, Yuichiro Tsuruta, Yuta Tsurusaki, Kohei Matsunaga, Shunsuke Tamanoi, Naoto Kuyama, Hiroki Usuku, Eiichiro Yamamoto, Yasuhiro Izumiya, Kenichi Tsujita

**Affiliations:** ^1^ Department of Cardiovascular Medicine, Graduate School of Medical Sciences Kumamoto University Kumamoto Japan; ^2^ Department of Cardiac Arrhythmias Kumamoto University Kumamoto Japan

**Keywords:** atrial arrhythmia, catheter ablation, implantable cardioverter defibrillator, pacemaker, transthyretin amyloid cardiomyopathy

## Abstract

In recent years, the number of cases diagnosed with wild‐type transthyretin amyloid cardiomyopathy (ATTRwt‐CM) has been increasing. However, ATTRwt‐CM frequently coexists with atrial fibrillation (AF), atrial flutter (AFL), and atrial tachycardia (AT), often necessitating management for arrhythmias. Additionally, ventricular arrhythmias sometimes occur, or conduction disturbances often develop, requiring management for bradycardia, frequently needing device therapy such as pacemakers, implantable cardioverter defibrillators (ICDs), or cardiac resynchronization therapy defibrillators. Therefore, for arrhythmia specialists, who primarily focus on non‐pharmacological treatments, arrhythmias associated with ATTRwt‐CM are unavoidable encounters, and becoming proficient in their management is increasingly important and considered essential. However, we sometimes encounter AF, AFL, or AT that are extremely difficult to treat with catheter ablation, and there are many situations to struggle with: how to manage each arrhythmia and whether catheter ablation should be performed at all. Furthermore, while the usefulness of ICDs for primary prevention of sudden cardiac death remains a subject of debate, we occasionally encounter patients with ventricular arrhythmias in fact. This review primarily addresses and focuses on catheter ablation therapy for atrial arrhythmias associated with ATTRwt‐CM, as well as device therapy for bradyarrhythmias and ventricular arrhythmias, aiming to provide insights for treatment planning in the future as a total management approach to arrhythmia in ATTRwt‐CM patients, especially for arrhythmia specialists.

## Graphical abstract

Transthyretin cardiomyopathy (ATTR‐CM) frequently coexists with atrial fibrillation, atrial flutter, and atrial tachycardia as well as occasionally ventricular arrhythmias and often conduction abnormalities, necessitating management for arrhythmias. Therefore, for arrhythmia specialists, the ability to diagnose ATTR‐CM and proficiency in arrhythmia management is increasingly important and considered essential. This review summarizes management approaches and indications for catheter ablation in various atrial arrhythmias associated with ATTR‐CM, as well as device therapy for bradyarrhythmias and ventricular arrhythmias, aiming to provide insights for treatment planning in the future as a total management approach to arrhythmia in ATTR‐CM patients, especially for arrhythmia specialists.

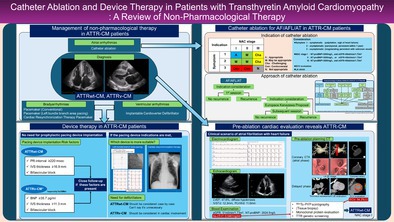

**Abbreviations**
AF: atrial fibrillation
AFL: atrial flutter
AL‐CM: immunoglobulin light chain type amyloid cardiomyopathy
AT: atrial tachycardia
ATTR‐CM: transthyretin amyloid cardiomyopathy
ATTRv‐CM: hereditary transthyretin amyloid cardiomyopathy
ATTRwt‐CM: wild‐type transthyretin amyloid cardiomyopathy
AV: atrioventricular
CTI: cavotricuspid isthmus
ICD: implantable cardioverter defibrillator
LBBAP: left bundle branch area pacing
PFA: pulse field ablation
SCD: sudden cardiac death
TTR: transthyretin

## 1 Introduction

In recent years, amyloid cardiomyopathy has garnered significant attention in the field of cardiology. Since it was reported that technetium 99 m pyrophosphate (^99m^Tc‐PYP) scintigraphy yields highly sensitive positive results specifically for transthyretin (TTR)‐related amyloid cardiomyopathy [1, 2], the widespread adoption of this diagnostic method has revealed that amyloid cardiomyopathy, particularly wild‐type transthyretin amyloid cardiomyopathy (ATTRwt‐CM), is more common than previously assumed and is encountered relatively frequently in daily clinical practice [3]. Furthermore, regarding treatment, in addition to advances in therapy for AL (amyloid light‐chain) amyloidosis, treatments for transthyretin amyloidosis are also rapidly progressing; the drug tafamidis, which stabilizes TTR tetramer and inhibits amyloid fiber formation and tissue deposition, has become a treatment for peripheral neuropathy in patients with hereditary transthyretin amyloidosis, and following the report of the ATTR‐ACT trial results [4], tafamidis has been used for both hereditary and wild‐type transthyretin amyloid cardiomyopathy (ATTR‐CM). Consequently, there has been an increase in cases screened for amyloid cardiomyopathy and subsequently treated.

**FIGURE 1:**
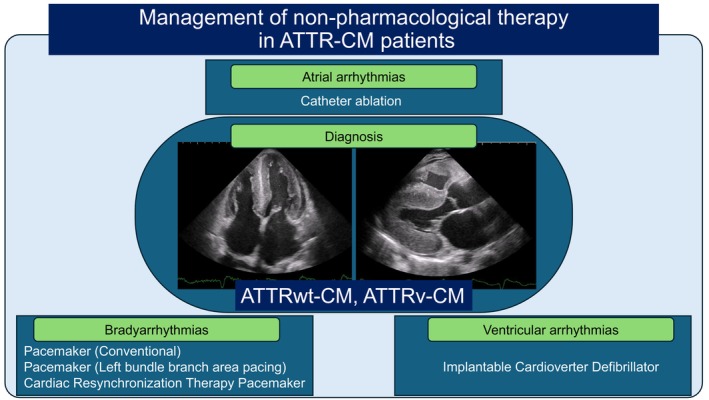
Management of non‐pharmacological therapy in ATTR‐CM patients. ATTR‐CM, transthyretin amyloid cardiomyopathy; ATTRv‐CM, hereditary transthyretin amyloid cardiomyopathy; ATTRwt‐CM, wild‐type transthyretin amyloid cardiomyopathy.

Meanwhile, ATTRwt‐CM occurs when wild‐type TTR, lacking genetic mutations, acts as an amyloid precursor protein and deposits throughout the body, but the direct impact of amyloid deposition in the atria, left atrial overload due to left ventricular diastolic dysfunction or increased left ventricular filling pressures, and the influence of patient high age contribute to the high prevalence of atrial fibrillation (AF), atrial flutter (AFL), and atrial tachycardia (AT) at a high frequency of 27%–67% in ATTRwt‐CM [5, 6, 7, 8, 9] and over 80% during follow‐up [10], and these arrhythmias have also been reported to be associated with heart failure [11]. However, in amyloid cardiomyopathy, due to the restrictive dysfunction, there is a tendency for reduced stroke volume and blood pressure; therefore, medications for heart failure such as angiotensin converting enzyme inhibitors, angiotensin II receptor blockers, beta‐blockers, and aldosterone antagonists should be administered with caution, and digitalis, verapamil, diltiazem, and other heart rate‐modulating agents, as well as antiarrhythmic drugs, should generally be avoided [12]. Furthermore, since pacemaker therapy has also been reported to worsen heart failure [13], when AF/AFL/AT coexists with amyloid cardiomyopathy, achieving sinus rhythm or managing heart rate control becomes challenging, and heart failure management is often extremely difficult. Therefore, consideration of catheter ablation as a non‐pharmacological treatment may be necessary (Figure 1), however, while pulmonary vein isolation (PVI) for AF generally has a high success rate of approximately 80%–90% in paroxysmal AF and is an established treatment, the efficacy of PVI for AF in the context of amyloid cardiomyopathy has been studied only in small numbers of patients. According to reports by Black‐Maier et al. and Barbhaiya et al. the 1‐year non‐recurrence rate was 17%–40%, significantly lower than the efficacy seen in typical paroxysmal AF [14, 15]. Furthermore, amyloid cardiopathy is known to frequently coexist with AT [14], and there is another report of the presence of multiple focal AT resistant to catheter ablation, a phenomenon not seen in other diseases [16]. However, in recent years, alongside an increase in patients diagnosed with ATTRwt‐CM, there has also been an increase in patients undergoing catheter ablation, and there are now reports indicating that the treatment outcomes of catheter ablation are not poor, except for some cases of multiple focal AT or complex AFL that show resistance to treatment [17]. This review summarizes previous reports on AF, AFL, and AT associated with ATTR‐CM—an extremely severe accumulative cardiomyopathy presenting with marked restrictive dysfunction—and aims to establish optimal treatment strategies for future clinical practice by explaining the usefulness of catheter ablation, its indications, and treatment methods.

Furthermore, another frequent issue in ATTR‐CM is the management of device therapy (Figure 1). While there are currently no consensus or guidelines providing indications for device implantation specifically in ATTR‐CM, it has been reported that the risk of fatal arrhythmias due to myocardial conduction disturbances caused by TTR deposition in the cardiac conduction system, as well as deposition in the ventricular myocardium and resulting myocardial damage, is higher than usual [18]. However, the utility of prophylactic pacemaker implantation remains unestablished, and clear indications for implantable cardioverter defibrillator (ICD) implantation as primary prevention for sudden cardiac death (SCD) are also absent. This review discusses the patient profile of ATTR‐CM requiring future pacing device implantation, the timing of implantation, the necessity of prophylactic implantation, and future perspectives on implantation techniques. In addition, it addresses the necessity of ICDs implantation as primary prevention for SCD based on the appropriate therapy.

Finally, it is most unfavorable that arrhythmia specialists treat AF/AFL/AT, bradyarrhythmias, or ventricular arrhythmias complicated to ATTR‐CM without recognizing the background disease in their daily practice. Although anyone would suspect some kind of cardiomyopathy in cases with advanced cardiac hypertrophy, it is often overlooked that ATTR‐CM could be present in cases with mild or less advanced left ventricular hypertrophy. Since it is not practical to view all patients with suspicion of ATTR‐CM, this review proposes relatively easy tips for identifying ATTR‐CM in daily practice.

## 2 Catheter Ablation for AF, AFL, and AT

Previous reports on catheter ablation for AF/AFL/AT in patients with amyloid cardiomyopathy (Table 1) include Black‐Maier et al.'s study of 10 patients (1 AL cardiomyopathy [AL‐CM], 9 ATTRwt‐CM) with persistent AF/AFL undergoing ablation. They reported recurrence in 6 (60%) at 1 year and 8 (80%) at 2 years, with non‐recurrence occurring in relatively younger patients [15]. Additionally, Donnellan et al. reported that among 24 AF patients (21 ATTRwt‐CM, 3 hereditary ATTR‐CM [ATTRv‐CM]), those with N‐terminal prohormone of brain natriuretic peptide (NT‐proBNP) > 3000 ng/L and estimated glomerular filtration rate (eGFR) < 45 mL/min/1.73m^2^ had a 3‐year recurrence rate of 9 in 10 patients, whereas among those not meeting these criteria, 9 in 14 patients did not have recurrence, demonstrating the efficacy of catheter ablation therapy in non‐progressive cases (Table 1) [19]. On the other hand, Barbhaiya et al. reported in a study of 18 patients (4 AL‐CM, 14 ATTRwt‐CM) that compared to non‐amyloidosis cases, left atrial mean voltage was reduced, AT was frequently induced, and the 1‐year recurrence rate was extremely poor at 83% (Table 1) [14]. Furthermore, in ATTRwt‐CM cases, the presence of multiple focal ATs is reported, which are induced and persist from multiple local origins, necessitating ablation of a total of eight focal ATs across two sessions, and even after that, ATs continued to be induced one after another, ultimately requiring atrioventricular (AV) node ablation and implantation of cardiac resynchronization therapy defibrillator (CRTD) [16]. Therefore, while catheter ablation for AF/AFL/AT in ATTRwt‐CM may be effective in some cases, it appears to be challenging in a significant number of patients, and its indications have been a subject of debate. Furthermore, while Mints et al. reported in 2018 that there was no significant difference in survival rates between patients with and without concomitant AF in ATTRwt‐CM [24], the ATTR‐ACT trial analysis indicated that AF remained an independent prognostic factor for all‐cause mortality even after adjusting for pre‐specified covariates (treatment, genotype, New York Heart Association [NYHA] functional class) [25]. In addition, Donnellan et al. reported that while no significant difference in mortality was observed between AF and non‐AF patients (65% vs. 49%; *p* = 0.76) during a mean follow‐up of 35 months, Cox proportional hazards analysis showed that maintaining normal sinus rhythm and using tafamidis were associated with improved survival [26]. Given recent improvements in catheter ablation outcomes and the introduction of tafamidis, these results may change.

**TABLE 1:** Characteristics of catheter ablation therapy for atrial arrhythmias in amyloid cardiomyopathy.

Study	Ablation patients number Target arrhythmias	Non‐recurrence rate	Characteristic findings
Black‐Maier 2020 [15]	AL‐CM 1, ATTRwt‐CM 9	40% (after 1 year)	Recurrence is more common in elderly patients
	Paroxysmal AF 2, Persistent AF 6, AFL 4	20% (after 2 years)	
Donnellan 2020 [19]	ATTRwt‐CM 21, ATTRv‐CM 3	64% (stage I, after 3 years)	Cases without progression in NAC stages I/II show fewer recurrences Ablation group had fewer hospitalizations for heart failure/arrhythmia (1.7 ± 2.4 vs. 4.0 ± 3.5 hospitalizations, *p* = 0.005) and improved prognosis
	Paroxysmal AF 4, Persistent AF 20	10% (stage III, after 3 years)	
Barbhaiya 2016 [14]	AL‐CM 4, ATTRwt‐CM 14	17% (after 1 year)	Recurrence rate after 1 year was 83% vs. 25% (HR: 5.4), significantly worse compared to the non‐amyloidosis group Left atrial voltage is reduced compared to non‐amyloidosis cases Atrial tachycardia was induced more frequently in ATTRwt‐CM (3.3 ± 1.9 ATs vs. 0.2 ± 0.4 ATs, *p* < 0.001)
	Persistent AF 18, AT 13		
Kanazawa 2024 [17]	ATTRwt‐CM 54	70.1% (after 1 year)	Catheter ablation significantly improved all‐cause mortality (HR: 0.342), cardiovascular mortality (HR: 0.378), and HF hospitalization (HR: 0.488) compared without catheter ablation Multiple focal AT and complex AFL showed extremely poor outcomes with catheter ablation, but the results for other arrhythmias were acceptable
	Paroxysmal AF 13, Persistent AF 29, CTI‐dependent AFL 24, non‐CTI dependent AFL 9, AT 18	57.6% (after 2 years)	
		44.0% (after 5 years)	
Tan 2016 [20]	AL‐CM 5, ATTRwt‐CM 7, ATTRv‐CM 1	75% (after 1 year)	AVN ablation in 13 patients (AL‐CM 2, ATTRwt‐CM 10, ATTRv‐CM 1) showed similar NYHA improvement compared to conventional ablation
	AF 5, AFL 8, AT 2	60% (after 3 years)	
Yakabe 2025 [21]	ATTRwt‐CM 53	78.4% (after 1 year)	Recurrence was lower and clinical outcomes were favorable in NAC stage I patients undergoing ablation Patients without recurrence had a lower incidence of the composite endpoint (heart failure hospitalization, ischemic stroke, and all‐cause mortality) (HR: 0.17)
	Paroxysmal AF 22, Persistent AF 31, Inducible AFL, AT (number unknown)	65.8% (after 2 years)	
Maury 2024 [22]	AL‐CM 12, ATTRwt‐CM 19	70% (after 1 year)	No recurrence after catheter ablation was associated with significant reductions in Cr and BNP and improvement in NYHA classification The non‐recurrence group had fewer heart failure hospitalizations, and recurrence was observed in all patients with heart failure deaths
	Paroxysmal AF 10, Persistent AF 12, AFL 17, AT 11	30% (after 2 year)	
Miyamoto 2023 [23]	AL 1, ATTR‐CM 8	75% (after 3 years)	AF was particularly common in patients with ATTR‐CM (70.0% in the ATTR‐CM vs. 23.1% in AL‐CM)
	AF8, AFL 1		

Abbreviations: AF, atrial fibrillation; AFL, atrial flutter; AL‐CM, immunoglobulin light chain type amyloid cardiomyopathy; AT, atrial tachycardia; ATTR‐CM, transthyretin amyloid cardiomyopathy; ATTRv‐CM, hereditary transthyretin amyloid cardiomyopathy; ATTRwt‐CM, wild‐type transthyretin amyloid cardiomyopathy; AVN, atrioventricular node; BNP, brain natriuretic peptide; Cr, creatinine; CTI, cavotricuspid isthmus; HR, hazard ratio; NAC, United Kingdom National Amyloidosis Centre; NYHA, New York Heart Association.

Amidst this context, a 2024 Europace published a study that compared 54 patients who underwent catheter ablation for AF/AFL/AT with 105 patients who did not, catheter ablation reduced the risk of hospitalization for heart failure (hazard ratio [HR]: 0.488; 95% confidence intervals [CI]: 0.269–0.889, *p* = 0.019), cardiovascular mortality (HR: 0.38; 95% CI: 0.146–0.981, *p* = 0.045), and all‐cause mortality (HR: 0.342; 95% CI: 0.133–0.876, *p* = 0.025) (Table 1) [17]. Within this study, the efficacy of catheter ablation was reported for each arrhythmia type—AF, AFL, and AT—including those responsive to catheter ablation and those resistant to that. Regarding cavotricuspid isthmus (CTI)‐dependent AFL, no recurrence of the same AFL was observed in all 24 cases, and even after checking CTI in 6 patients who underwent multiple sessions for other arrhythmias, no CTI recurrence was seen in any case. Therefore, catheter ablation is recommended for CTI‐dependent AFL. Furthermore, regarding paroxysmal AF and persistent AF, thorough PVI fundamentally prevented recurrence as AF in 12 of 13 paroxysmal AF cases and 27 of 29 persistent AF cases, which is a remarkable result. Conversely, regarding non‐CTI dependent AFL, though simple AFL cases where atrial voltage is sufficiently maintained to allow mapping and treatable with single‐line ablation—such as mitral AFL or LA anterior channel dependent AFL—were manageable like non‐ATTR‐CM patients with low recurrence, complex AFL with low atrial voltage, difficult circuit identification, often failing to terminate despite multiple ablation sites, and even when AFL is successfully terminated and becomes non‐inducible, recurrence or new circuit formation frequently occurs. Regarding AT, it has been reported that for AT originating from the crista terminalis or the AV annulus, ablation successfully terminated the AT and was considered curable, similar to patients without ATTR‐CM, whereas for multiple focal AT primarily occurring in the right atrial septum, near the coronary sinus ostium, or the right atrial appendage, no matter how many times ablation was performed, the focus would simply shift to another region, making termination extremely difficult and resulting in a very high recurrence rate. It truly resembles a “wandering AT”, and while some paroxysmal AT episodes may become less frequent, persistent AT almost invariably recurs during follow‐up, considering it highly troublesome.

However, as this paper demonstrates, catheter ablation for ATTR‐CM has proven effective for most cases except complex AFL and multiple focal AT, which show resistance to treatment, and consequently, reductions in heart failure hospitalizations, cardiovascular mortality, and overall mortality can be expected; therefore, catheter ablation is fundamentally considered useful. Whereas complex AFL and multiple focal AT were reported to occur more frequently in cases with cardiac magnetic resonance imaging quantified extracellular volume (MRI‐ECV) exceeding 60%, suggesting that troublesome arrhythmias are indeed more likely to occur in advanced ATTR‐CM. The non‐recurrence rate for overall AF/AFL/AT outcomes after a single ablation procedure was 61.3% at 1 year, 50.2% at 2 years, and 27.4% at 5 years, while the rates after multiple treatments were 70.1% at 1 year, 57.6% at 2 years, and 44.0% at 5 years (Table 1), meaning that in a sense, treatment outcomes do not improve significantly even with multiple procedures. Conversely, this suggests that the determining factor for treatment outcomes is the hidden presence of multiple focal ATs, and since their induction was the predictive factor for recurrence, even repeated treatments may yield little improvement once multiple focal ATs emerge. Given this, catheter ablation outcomes in advanced cases may not be highly promising.

**FIGURE 2:**
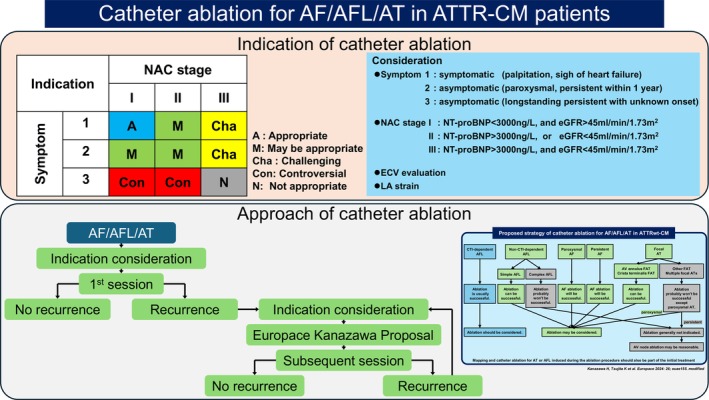
Catheter ablation for AF/AFL/AT in ATTR‐CM patients. Indications and approach for catheter ablation in AF/AFL/AT cases with ATTR‐CM. AF, atrial fibrillation; AFL, atrial flutter; AT, atrial tachycardia; ATTR‐CM, transthyretin amyloid cardiomyopathy; ATTRwt‐CM, wild‐type transthyretin amyloid cardiomyopathy; AV, atrioventricular; CTI, cavotricuspid isthmus; ECV, extracellular volume; eGFR, estimated glomerular filtration rate; FAT, focal atrial tachycardia; LA, left atrial; NAC, United Kingdom National Amyloidosis Centre; NT‐proBNP, *N*‐terminal prohormone of brain natriuretic peptide.

In this paper, catheter ablation was performed for symptomatic AF/AFL/AT presenting with palpitations or signs of heart failure, or for asymptomatic AF/AFL/AT occurring within 1 year of onset. Even if advanced, catheter ablation may be worth attempting once for AF/AFL/AT causing heart failure or accompanied by palpitations (Figure 2). Consequently, if the AFL is CTI‐dependent, curative ablation is possible, and heart failure control may improve dramatically. However, in cases of multiple focal AT or complex AFL, careful consideration of indications is warranted for second and subsequent sessions. While performing ablation is certainly possible, the outcome may be similar; if unavoidable, AV node ablation plus device implantation should also be considered. Tan et al. reported that, while the 1‐year and 3‐year recurrence‐free survival rates in 13 cases (5 AL‐CM, 7 ATTRwt‐CM, 1 ATTRv‐CM) who underwent catheter ablation for atrial arrhythmias (5 AF, 8 AFL, 2 AT) were 75% and 60%, respectively, in 13 patients who underwent AV node ablation (2 AL‐CM, 10 ATTRwt‐CM, 1 ATTRv‐CM), NYHA class improvement was comparable to that in 13 patients treated with catheter ablation, suggesting AV node ablation is a reasonable option even in advanced amyloidosis cases [20]. On the other hand, in less advanced cases, multiple focal AT or complex AFL are less likely to occur. A similar report to Donnellan et al. [19] was recently published by Yakabe et al., indicating fewer recurrences in the United Kingdom National Amyloidosis Centre (NAC) Stage I (NT‐proBNP < 3000 ng/L and eGFR > 45 mL/min1.73m^2^) [27] (HR: 0.21, 95% CI: 0.08–0.56, *p* = 0.002) (Table 1) [21]. Catheter ablation seems undoubtedly useful in early‐stage ATTR‐CM cases (Figure 2), and given recent trends, early diagnosis and treatment of ATTR‐CM are considered increasingly important. Furthermore, several other reports have emerged regarding the usefulness of catheter ablation for ATTR‐CM (Table 1) [22, 23]. All of these studies provide data supporting ablation's efficacy, and meta‐analyses also reach similar conclusions [28]. Additionally, another report has emerged indicating that catheter ablation for AF in patients with amyloid cardiomyopathy was associated with improvements in overall mortality (odds ratio [OR]: 0.404; 95% CI: 0.182–0.896) and hospitalization rates (OR: 0.547; 95% CI: 0.317–0.942) compared to antiarrhythmic drug therapy [29]. Catheter ablation for ATTR‐CM is likely to be effective except in cases of multiple focal AT or complex AFL, and it may be advisable to aggressively consider catheter ablation for patients suffering from heart failure or palpitations, as well as persistent cases within approximately 1 year of onset, with the expectation of improving heart failure hospitalization and prognosis (Figure 2). On the other hand, it is uncertain whether ablation is useful in cases of AF/AFL/AT in which the onset time is unknown, heart failure is well compensated, and the patient experiences no symptoms. Indeed, in such cases, AF may not be a prognostic factor [24], and establishing the utility of ablation for these patients remains challenging because the treatment outcomes for long‐standing AF/AFL/AT may inherently be poor. Considering the prognosis of ATTR‐CM itself, it is difficult to establish informed consent justifying the value of performing catheter ablation and how aggressively intervening in patients with AF/AFL/AT of unknown onset who are not experiencing symptoms.

Finally, pulse field ablation (PFA) for ATTR‐CM remains an area of interest for the future. While PFA for atrial fibrillation has rapidly gained widespread adoption and its utility is becoming established [30], all previous reports on catheter ablation for ATTR‐CM have involved radiofrequency ablation, and according to the Europace report [17], catheter ablation was performed using moderate‐power with index‐guide ablation (25‐35 W, lesion size index ≥ 5.2 or ablation index ≥ 400). Some reports may have used high power short duration therapy, but in amyloidosis, the atrial wall may be thicker than normal, and using moderate‐power with index‐guide ablation therapy might have resulted in higher quality PVI. Conversely, as PFA becomes more common, amidst concerns that lesion depth may be shallow without proper contact [31] or PFA lesions are more inhomogeneous compared to radiofrequency ablation [32], the PV durability of PFA for ATTR‐CM will likely become clearer in the future. Since AFL or AT frequently coexist in ATTR‐CM, whether radiofrequency catheter ablation or PFA is the optimal treatment device for ATTR‐CM cases is another issue expected to be clarified by future research.

## 3 Pacing Device Therapy for Bradyarrhythmias

**FIGURE 3:**
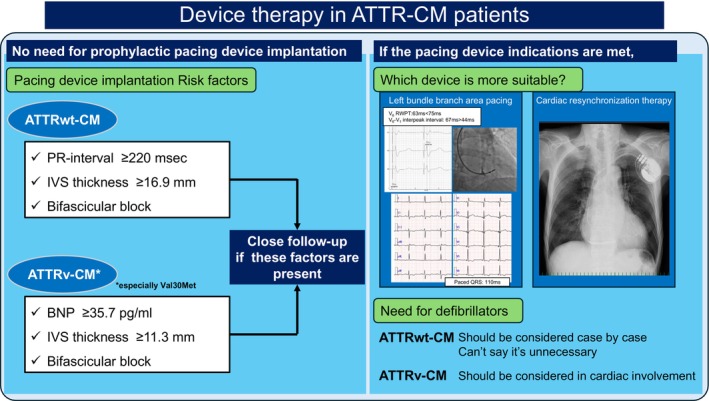
Device therapy in ATTR‐CM patients. Patient profiles requiring careful follow‐up, pacing methods, and ICD necessity when a device becomes necessary in ATTR‐CM patients. ATTR‐CM, transthyretin amyloid cardiomyopathy; ATTRv‐CM, hereditary transthyretin amyloid cardiomyopathy; ATTRwt‐CM, wild‐type transthyretin amyloid cardiomyopathy; IVS, interventricular septal; RWPT, *R*‐wave peak time.

Previous reports have demonstrated the utility of prophylactic pacemaker implantation in ATTRv‐CM [33]. In 100 patients meeting criteria: HV interval ≥ 70 msec, HV interval ≥ 55 msec with fascicular block, HV interval ≥ 55 msec with PR interval ≥ 200 msec, or HV interval ≥ 55 msec with wenckebach point ≤ 100 beat/min, prophylactic pacemaker implantation was performed, resulting in progression to high‐degree AV block in 25% at 5 years and identifying the following risk factors: first‐degree AV block (PR interval ≥ 200 msec), wenckebach anterograde points ≤ 100 beats/min, and absence of microvoltage on surface electrocardiogram. In general, pacemaker implantation is not indicated for first‐degree AV block, but based on this report, many neurologists began requesting prophylactic pacemaker implantation to cardiologists, and many cardiologists were confused. Actually, even now, there are no guidelines or consensus statements recommending prophylactic pacemaker implantation specifically for ATTR‐CM, and implantation must be performed in accordance with American heart association, European society of cardiology, and Japanese circulation society guidelines, just as with standard pacemaker implantation indications. Certainly, it is known that conduction disturbances are frequently observed in ATTR‐CM due to TTR deposition in the cardiac conduction system. In ATTRwt‐CM, it has been reported that TTR deposition tends to occur in a pericellular pattern and nodular pattern [34], and ultimately spreads diffusely [35], affecting the cardiac conduction system and causing AV block and intraventricular conduction block. On the other hand, previous studies have demonstrated direct amyloid deposition in the sinus node and conduction system of ATTRv‐CM patients [36, 37]. Furthermore, another study demonstrated that amyloid deposition in the subendocardial myocardium (including the conduction system) in young‐onset ATTRv‐CM patients with the Val30Met mutation causes a high incidence of subendocardial myocardial atrophy and conduction abnormalities, while late‐onset ATTRv‐CM patients had diffuse amyloid deposition [38]. Therefore, it is clear that ATTR‐CM is highly associated with conduction disturbances, with the prevalence approximately 11%–33% in ATTRwt‐CM and 25%–45% in ATTRv‐CM [6, 7, 12, 39, 40]. Furthermore, cases of ATTRwt‐CM presenting with LBBB conduction abnormalities alongside increased heart rate have been reported [41]. In this context, identifying predictive factors for future pacemaker implantation would be useful for careful follow‐up, thus a study was conducted to investigate these factors [40]. According to this research, in ATTRwt‐CM, a PR interval ≥ 220 msec, interventricular septal (IVS) thickness ≥ 16.9 mm, and bifascicular block are predictive factors leading to future pacemaker implantation. Similarly, in ATTRv‐CM, brain natriuretic peptide (BNP) ≥ 35.7 pg/mL, IVS thickness ≥ 11.3 mm, and bifascicular block are predictive factors (Figure 3). Notably, simply having first‐degree AV block alone does not require future pacemaker implantation in either ATTRwt‐CM or ATTRv‐CM. In this study, val30met accounted for 72% of cases, therefore, even in cases where TTR does not accumulate in the whole heart but accumulates directly in the conduction system leading to pacemaker implantation, slight changes like BNP ≥ 35.7 pg/mL and IVS thickness ≥ 11.3 mm may indicate TTR accumulation in the heart and suggest the need for attention to the conduction disturbances. Conversely, in ATTRwt‐CM, TTR accumulates diffusely, thus conduction disturbances likely occur at a more advanced stage, as indicated by IVS thickness ≥ 16.9 mm. Interestingly, BNP or high‐sensitivity cardiac troponin T (hs‐cTnT) levels cannot predict pacemaker implantation, with bifascicular block being the strongest predictor in ATTRwt‐CM. In clinical practice, bifascicular block is frequently observed in ATTRwt‐CM, and it is useful information at the time of informed consent that patients with bifascicular block have a particularly high likelihood of requiring pacemaker implantation in the future. However, there is currently no data demonstrating the benefit of proactively implanting pacemakers in patients with these identified predictors, so the utility of prophylactic pacemaker implantation in these patients remains unclear. There are even reports indicating that pacing may worsen cardiac function [13], therefore, at present, implanting a pacemaker should be considered appropriate only when the indications described in the guidelines are met (Figure 3).

Regarding implantation technique, numerous challenges have been undertaken recently to determine whether conventional pacemakers, cardiac resynchronization therapy (CRT), or left bundle branch area pacing (LBBAP) is preferable (Figure 3). In amyloidosis, the left ventricular ejection fraction is often reduced to < 50%, and CRT or LBBAP is frequently preferable to simple ventricular pacing, similar to the BLOCK HF study [42]. In such cases, at least leadless pacemakers are unlikely to be recommended. CRT is generally considered appropriate based on several pieces of evidence, but since ATTR‐CM patients are often elderly, the complexity of the procedure is sometimes viewed unfavorably. Therefore, LBBAP is increasingly replacing CRT. However, ATTR‐CM cases present the issue of whether LBBAP is even possible due to the thickened interventricular septum, but case report [43] and case report series [44] regarding LBBAP in ATTR‐CM are beginning to be published, suggesting LBBAP might be relatively successful. In addition, since ATTR‐CM patients are elderly and the expected lifespan after implantation is not considered particularly long, the issue of whether lead extraction will be possible is unlikely to arise frequently. When implanting devices in ATTR‐CM patients, the question arises whether LBBAP alone is sufficient because it also involves the issue of the necessity of ICDs discussed next; however, as shock leads for LBBAP are expected to become available in the future, this problem is also expected to be resolved.

## 4 The Need for ICD Implantation for Ventricular Arrhythmias

Regarding the rate of appropriate ICD therapy, Lin et al. reported it was observed in 15 of 53 amyloidosis patients (28%) with AL‐CM showing a higher rate of 12 in 33 patients (36%) compared to ATTRwt‐CM (2 in 10 patients, 20%) and ATTRv‐CM (0 in 9 patients, 0%) (Table 2) [45]. Similarly, Varr et al. reported appropriate ICD therapy in 5 of 19 (26%) amyloidosis patients, with 5 of 15 (33%) AL‐CM patients experiencing appropriate ICD therapy but no appropriate therapy in either the 2 ATTRwt‐CM or 2 ATTRv‐CM patients (Table 2) [46]. In Kim et al.'s report where all patients received ICDs implantation for primary prevention of SCD, appropriate ICD therapy occurred in 2 of 7 (29%) AL‐CM patients, 1 of 10 (10%) ATTRwt‐CM patients, and 2 of 6 (33%) ATTRv‐CM patients [47], suggesting that even in primary prevention, AL‐CM appears to have a higher appropriate therapy rate than ATTR‐CM, but that appropriate therapy is not absolutely zero even in primary prevention ATTR‐CM patients (Table 2). Furthermore, in the report by Hamon et al., although the details of the patient subgroup are unclear, appropriate ICD therapy was observed in 12 of 45 patients (27%), indicating that a certain proportion of patients with amyloidosis do experience appropriate ICD therapy [48]. Dale et al. reported an appropriate ICD therapy rate of 2 of 19 patients (11%) in their study focusing solely on ATTRwt‐CM, suggesting a lower rate than in AL‐CM, similar to reports by Lin and Varr et al., though it was still not zero (Table 2) [49]. Furthermore, Kawahara et al. reported the appropriate ICD activation rate in ATTR‐CM from Japan: 2 of 16 cases (13%) in ATTRwt‐CM and 1 of 3 cases (33%) in ATTRv‐CM (Table 2) [40]. Similar to previous reports, the appropriate ICD therapy rate in ATTR‐CM may not be as high as in AL‐CM, but in Kawahara et al.'s report, both of the 2 ATTRwt‐CM patients who experienced appropriate ICD therapy were those implanted with ICDs for primary prevention, whereas the 1 patient implanted with an ICD for secondary prevention did not experience appropriate therapy, despite being on oral amiodarone therapy. Furthermore, the one ATTRv‐CM patient with appropriate therapy was also an ICD implant patient for primary prevention, raising the question of which patients require primary prevention ICD implantation. Brown et al. also reported that ICDs were implanted as primary prevention in 7 cases of ATTRwt‐CM and 25 cases of ATTRv‐CM, with appropriate therapy observed in 8 cases (2 ATTRwt‐CM [28%], 6 ATTRv‐CM [24%]) [50]. In AL‐CM, it has been reported that prophylactic ICD implantation may be proposed for patients with particularly wide QRS duration or bundle branch block/interventricular conduction delay, aiming to improve survival in advanced cases [51]. On the other hand, there are also reports indicating that appropriate ICD therapy in amyloid cardiomyopathy is not predicted by non‐sustained ventricular tachycardia [52]. Indeed, there are no clear guidelines or consensus recommending ICD implantation for primary prevention in amyloidosis currently, and previous 2013 appropriate use criteria for ICD and CRT only stated “maybe appropriate” for amyloidosis with heart failure, regardless of ejection fraction [53], consistent with Kawahara et al.'s findings that ejection fraction in their ATTRwt‐CM cases with appropriate ICD therapy for primary prevention was 50.3% and 40.7%, both above 35% [40]. In summary, while the rate of appropriate ICD therapy for ventricular tachycardia/ventricular fibrillation may be lower with ATTR‐CM than with AL‐CM, it is not entirely absent in ATTR‐CM (Figure 3). Since we sometimes encounter an ATTRwt‐CM patient who occasionally experiences ventricular arrhythmias and requires ICD implantation for secondary prevention, it is advisable to thoroughly evaluate the ICD function for primary prevention on a case‐by‐case basis especially when pacing devices are considered to implant even in elderly patients (Figure 3). A mechanism suggesting a lower rate of appropriate ICD therapy in ATTR‐CM compared to AL‐CM may be the difference in the pattern of amyloid deposition. In AL‐CM, amyloid deposition can occur in a focal patchy pattern, whereas in ATTRwt‐CM, global diffuse amyloid deposition occurs in all cases [35]. In AL‐CM, healthy myocardium coexists with amyloid‐deposited myocardium, potentially creating areas of conduction delay in the amyloid‐deposited tissue where reentry can initiate. Conversely, in ATTRwt‐CM, the diffuse amyloid deposition leaves little healthy tissue, which might actually make reentry less likely. However, ATTR‐CM also develops areas of conduction delay throughout the myocardium, and reentry can certainly occur, meaning ventricular arrhythmias are still possible. Therefore, Lin et al.'s report shows that in AL‐CM, appropriate ICD therapy did occur subsequently in 6 out of 8 cases (75%) for secondary prevention and in 6 of 25 cases (24%) for primary prevention, while in ATTRwt‐CM, appropriate therapy was not observed for primary prevention but did occur thereafter in both of the 2 cases for secondary prevention [45], clearly indicating the necessity of ICD implantation for secondary prevention (Table 2). Varr et al. also reported, in AL‐CM, appropriate ICD therapy in 3 of 4 cases (75%) for secondary prevention and in 2 of 11 cases (18%) for primary prevention [46]. Dale's report similarly shows appropriate activation in 1 of 1 patient for secondary prevention and in 1 of 18 patients for primary prevention in ATTRwt‐CM [49], as well as in reports by Kim et al. [47] and Brown et al. [50], suggesting that the necessity for ICD implantation for primary prevention of SCD, particularly in amyloidosis and especially ATTR‐CM, cannot yet be definitively denied. Recently, there is a trend toward prioritizing LBBAP, while the shock function is being neglected with concern. The necessity of ICDs for primary prevention should be discussed beyond just EF, and further consideration is needed in the future (Figure 3).

**TABLE 2:** Characteristics of the ICD study in amyloid cardiomyopathy.

Study		Total			AL‐CM			ATTRwt‐CM			ATTRv‐CM		
		Total	Primary prevention	Secondary prevention	Total	Primary prevention	Secondary prevention	Total	Primary prevention	Secondary prevention	Total	Primary prevention	Secondary prevention
Lin 2013 [45]	Patient number, *n*	53	41	12	33	25	8	10	8	2	9	7	2
	Appropriate ICD therapy, *n* (%)	15 (28)	7 (17)	8 (75)	12 (36)	6 (24)	6 (75)	2 (20)	0 (0)	2 (100)	0 (0)	0 (0)	0 (0)
Varr 2014 [46]	Patient number, *n*	19	15	4	15	11	4	2	2	0	2	2 (2 Val122Ile)	0
	Appropriate ICD therapy, *n* (%)	5 (26)	2 (13)	3 (75)	5 (33)	2 (18)	3 (75)	0 (0)	0 (0)	—	0 (0)	0 (0)	—
Kim 2020 [47]	Patient number, *n*	23	23	0	7	7	0	10	10	0	6	6	0
	Appropriate ICD therapy, *n* (%)	5 (22)	5 (22)	—	2 (29)	2 (29)	—	1 (10)	1 (10)	—	2 (33)	2 (33)	—
Hamon 2016 [48]	Patient number, *n*	45	38	7	12			6			27		
	Appropriate ICD therapy, *n* (%)	12 (27)	10 (26)	2 (29)									
Dale 2022 [49]	Patient number, *n*	19	18	1				19	18	1			
	Appropriate ICD therapy, *n* (%)	2 (11)	1 (6)	1 (100)				2 (11)	1 (6)	1 (100)			
Kawahara 2023 [40]	Patient number, *n*	19	17	2				16	14	2	3	3	0
	Appropriate ICD therapy, *n* (%)	3 (16)	3 (18)	0 (0)				2 (13)	2 (14)	0 (0)	1 (33)	1 (33)	—
Brown 2022 [50]	Patient number, *n*	32	32	0				7	7	0	25	25	0
	Appropriate ICD therapy, *n* (%)	8 (25)	8 (25)	—				2 (28)	2 (28)	—	6 (24)	6 (24)	—

*Note:* Other abbreviations as in Table 1.

Abbreviation: ICD, implantable cardioverter defibrillator.

On the other hand, in ATTRv‐CM, Kawahara et al. reported that the only patient with appropriate ICD therapy had the Thr60Ala mutation, while the remaining two patients without appropriate ICD activation had the Val30Met mutation [40]. The Thr60Ala mutation is associated with a higher likelihood of cardiac complications and poorer prognosis compared to Val30Met [54]. Furthermore, in Brown et al.'s report of 8 appropriate ICD therapy patients in 32 (7 ATTRwt‐CM, 25 ATTRv‐CM) ICD implanted cases for primary prevention, 89% of ATTRv‐CM cases carried the Val122Ile mutation [50]. In addition, Val30Met mutations exhibit different characteristics depending on whether it is an early‐onset case from an endemic area or a late‐onset case from a non‐endemic area [55]. The appropriate ICD therapy rate in ATTRv‐CM is likely to vary depending on the genetic mutation and the early‐ or late‐onset forms; therefore, ICD implantation for primary prevention of SCD should be adequately considered, at least for ATTRv‐CM patients with cardiac involvement (Figure 3).

## 5 Early Diagnosis and Early Treatment of Amyloid Cardiomyopathy in Arrhythmia Management

To improve treatment outcomes and prognosis for these arrhythmias, therapeutic approaches targeting amyloidosis itself are also critically important. Donnellan et al. reported that patients who took tafamidis after catheter ablation maintained sinus rhythm more frequently compared to those who did not take it (32% vs. 10%, *p* = 0.001) [26]. Furthermore, Girvin et al. reported that taking tafamidis suppressed the new onset of AF itself by 57% [56]. On the other hand, Isotani et al. reported that AF persisting for approximately 1 year recovered to normal sinus rhythm within a few months after starting tafamidis, without the need for antiarrhythmic drugs [57]. In addition, Nishizawa et al. showed that while left atrial function (LA reservoir strain) did not improve with tafamidis in AF patients, it improved (10.5% ± 5.0% to 11.9% ± 5.3%, *p* = 0.0307) in sinus rhythm patients taking tafamidis [58]. Ichikawa et al. demonstrated that tafamidis suppressed the worsening of echocardiographic parameters at an average of 16 ± 8 months after administration compared to pre‐treatment levels, and although not statistically significant, left atrial volume (LA volume index) showed a trend toward reduction (53.0 ± 17.7 vs. 49.0 ± 17.1, *p* = 0.299) [59]. A decline in left atrial function (left atrial strain reservoir: LAS_R_) has also been reported to increase the recurrence rate of AF after PVI (HR 0.86; 95% CI 0.73–1.00; *p* = 0.046) [60], indicating that taking tafamidis may prevent worsening or improve left atrial function, and consequently, may be expected to prevent the onset of atrial fibrillation.

**FIGURE 4:**
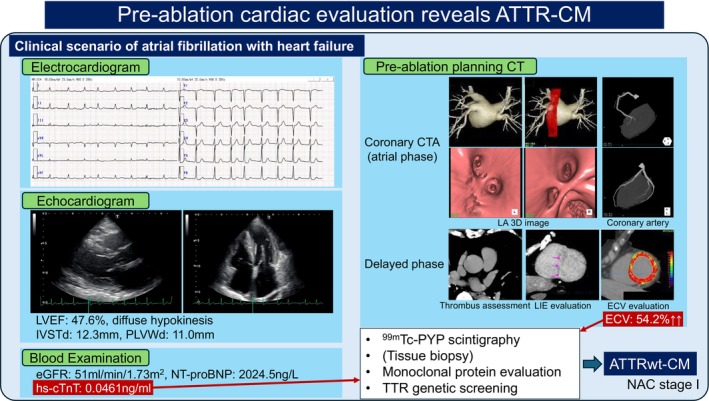
Pre‐ablation cardiac evaluation reveals ATTR‐CM. Clinical scenario of atrial fibrillation with heart failure is shown in this slide. ATTR‐CM, transthyretin amyloid cardiomyopathy; ATTRwt‐CM, wild‐type transthyretin amyloid cardiomyopathy; CT, computed tomography; CTA, CT angiography; ECV, extracellular volume; eGFR, estimated glomerular filtration rate; hs‐cTnT, high‐sensitivity cardiac troponin T; IVSTd, interventricular septal wall thickness at end‐diastole; LA, left atrium; LIE, late iodine enhancement; LVEF, left ventricular ejection fraction; NT‐proBNP, *N*‐terminal prohormone of brain natriuretic peptide; PLVWd, posterior left ventricular wall thickness at end‐diastole; TTR, transthyretin; 3D, three‐dimensional; ^99m^Tc‐PYP, technetium 99 m pyrophosphate.

Furthermore, as reported by Donnellan et al. [19] and Yakabe et al. [21], recurrence after catheter ablation for AF associated with ATTR‐CM is less frequent in cases with lower NAC stages [27] that have not yet progressed, compared to cases with higher NAC stages that have advanced. In other words, recurrence rates become extremely high in advanced cases, making early‐stage disease detection and therapeutic approaches critically important (Figures 2 and 4). For arrhythmia specialists, there are several opportunities to screen for amyloidosis. First, following the red flags algorithm [61], ATTR‐CM is suspected in patients with, for example, a left ventricular wall thickness of 12 mm who also present with heart failure, low QRS voltage, or pseudo‐infarction patterns; however, it is difficult to suspect ATTR‐CM based solely on a wall thickness around 12 mm, and the sensitivity of low QRS voltage or pseudo‐infarction patterns is not particularly high [62], making early screening for ATTR‐CM challenging with these criteria alone (Figure 4). On the other hand, when hs‐cTnT is 0.03 ng/mL or higher, 9.1% of cases led to a diagnosis of ATTR‐CM [63], suggesting that routinely measuring hs‐cTnT could be one approach to initiate diagnosis (Figure 4). Elevated hs‐cTnT is a poor prognostic factor, and tafamidis has been shown to suppress the progression of elevated hs‐cTnT and BNP [64]. Meanwhile, it has also been reported that patients who developed composite events did not decrease their hs‐cTnT or BNP levels even while taking tafamidis [65], suggesting hs‐cTnT is also important for assessing disease progression. Furthermore, ATTR‐CM often coexists with carpal tunnel syndrome (reported in 30%–45% of cases) [5, 6, 39, 66] and spinal canal stenosis (reported in 22% of ATTRwt‐CM cases) [66, 67], but it is not practical for arrhythmia specialists to systematically screen these two conditions in every patient referred for arrhythmia treatment. Nevertheless, the coexistence of carpal tunnel syndrome or spinal canal stenosis can also serve as a hint suggesting the possibility of ATTR‐CM. Therefore, if such comorbidity is present, it is important to check for ATTR‐CM with a suspicious eye. Conversely, it has been reported that amyloid deposition was present in 34%–37%, and ATTRwt‐CM was already coexisting in 5%–19% of cases who underwent surgery for carpal tunnel syndrome [68, 69], suggesting that collaboration with orthopedic surgery within the hospital is also important [70], because carpal tunnel syndrome and spinal canal stenosis tend to precede cardiac involvement in ATTRwt‐CM patients [10], potentially enabling ultra‐early diagnosis of ATTRwt‐CM. Furthermore, performing ^99m^Tc‐PYP scintigraphy based on the Kumamoto Criteria [71], or combining it with hs‐cTnT as a clue [72], may also be useful. The Kumamoto Criteria includes hs‐cTnT ≥ 0.0308 ng/mL, LV posterior wall thickness ≥ 13.6 mm, and wide QRS (QRS width ≥ 120 ms), and 3 of these 3 factors yield a ^99m^Tc‐PYP scintigraphy positivity rate of 96%, while 2 out of 3 yield 63%. However, it may be difficult to recognize these factors without routinely suspecting amyloidosis. Therefore, for arrhythmia specialists, the simplest and most noticeable method for suspecting ATTRwt‐CM in daily practice seems to be ECV quantified by computed tomography (CT‐ECV) (Figure 4). Normal ECV is approximately 25% and increases in conditions such as reactive fibrosis (hypertension, valvular heart disease, diabetes), replacement/scarring fibrosis (myocardial infarction, myocarditis, sarcoidosis), or infiltrative fibrosis (amyloidosis, Fabry disease) [73]. In particular, amyloidosis is associated with the most pronounced increase in ECV, reaching at least 35% in ATTR‐CM [74] and potentially as high as 80%–90% in advanced cases [16]. Using contrast‐enhanced CT (such as coronary CT angiography, pre‐transcatheter aortic valve replacement planning cardiac CT, and pre‐atrial fibrillation ablation planning cardiac CT) to assess ECV, it has been reported that amyloid cardiomyopathy could be screened with a sensitivity of 90% and specificity of 92% at an ECV of 37% [75], even focusing solely on coronary CT, it was reported that amyloid cardiomyopathy coexisted in as many as 14.3% of patients with an ECV exceeding 35% [76]. Since a case report described a patient with ECV as high as 65% on pre‐ablation planning CT, leading to ^99m^Tc‐PYP scintigraphy and ultimately a diagnosis of ATTRwt‐CM [77], it is expected that evaluating ECV on contrast‐enhanced CT before ablation will significantly improve the diagnostic rate of amyloidosis (Figure 4). However, ECV measurement requires delayed‐phase imaging, which necessitates increased radiation exposure and the addition of a small amount of contrast agent [78]. Therefore, it has been reported that limiting delayed‐phase imaging and ECV measurement to patients with positive red flag findings can be useful for diagnosing ATTR‐CM [79]. However, recent advances in CT technology have significantly reduced radiation exposure, such as using a 320‐slice flat panel detector, limiting exposure to the diastolic phase only, controlling heart rate with beta‐blockers to fit the exposure within a single diastolic beat, and employing new deep learning image reconstruction techniques (which tolerate slightly poorer raw image quality). Combining plain CT, coronary CT, and delayed phase imaging can limit the effective dose to approximately 2 mSv per scan, totaling 6 mSv—equivalent to the typical exposure from a plain chest CT scan. While it may not be possible to say there is absolutely no radiation exposure risk, there is not enough evidence to suggest any risk, especially in elderly patients. For patients undergoing arrhythmia treatment, it is reasonable to perform CT‐ECV evaluation in all possible patients as part of the thorough assessment of underlying conditions, not limited to amyloidosis under the principle of medical radiation exposure justification (careful consideration of indications) and optimization (parameter adjustment) (Figure 4). Furthermore, evidence supporting contrast‐induced nephropathy (CIN) from intravenous administration has been increasingly refuted over the years. Multiple studies report that contrast‐enhanced CT does not pose a CIN risk even in patients with an eGFR below 30 mL/min/1.73m^2^ [80], suggesting that the risk of CIN from contrast‐enhanced CT is low in patients with an eGFR of 30 mL/min/1.73m^2^ or higher. Since the amount of contrast agent added during the delayed phase is approximately 100 mg iodine/kg, which translates to about 16 mL for a 60 kg patient (370 μg iodine), excessive restriction of delayed‐phase contrast enhancement should not be considered from a contrast agent perspective. Contrast‐enhanced CT before catheter ablation is routinely performed by arrhythmia specialists for purposes such as evaluating the morphology of the pulmonary veins and left atrium, assessing vascular access, and checking for intracardiac thrombi (Figure 4). Routinely adding delayed‐phase imaging not only allows for thrombus detection with a higher negative predictive value but also enables thorough evaluation of underlying cardiac disease, consequently naturally identifying cases of amyloid cardiomyopathy. Once diagnosed with ATTR‐CM, disease‐modifying therapies such as tafamidis can be initiated. Therefore, it is expected that arrhythmia specialists will increasingly detect and treat ATTR‐CM in the future. Minamisawa et al. reported that in ATTR‐CM, complications suggesting disease onset included heart failure (87.9%), AF/AFL (50.2%), and conduction disturbances (17.2%), and the median time from onset of each complication to ATTR‐CM diagnosis and initiation of treatment was 15.5 months, 14.0 months, and 9.0 months, respectively [9]. It is impossible not to hope that this period will become increasingly shorter following the onset of arrhythmia.

## 6 Conclusion

A significant number of amyloid cardiomyopathy patients undergo non‐pharmacological treatments such as catheter ablation or device therapy. Particularly in ATTR‐CM, we sometimes encounter challenging arrhythmias like multiple focal AT or complex AFL; therefore, it is critically important to recognize ATTR‐CM as the underlying cardiac disorder and understand the arrhythmias as complications of this disease. Knowing ATTR‐CM prior to catheter ablation facilitates the development of a treatment strategy tailored to each arrhythmia encountered. In this regard, pre‐ablation disease screening and staging assessment using troponin T and CT‐ECV appear to be critically important. Similarly, for device therapy, if conduction abnormalities are superimposed on ATTR‐CM, LBBAP, ICD implantation for primary prevention of SCD, and potentially CRTD may be preferable. Those involved in non‐pharmacological treatment of arrhythmias should serve as the commander of total management, focusing not only on the arrhythmia itself but also on thorough screening and treatment of the patient's background.

## Funding

This work was supported by JSPS KAKENHI, 23K07580.

## Consent

The authors have nothing to report.

## Conflicts of Interest

H.K. has received grants from Medtronic Japan, Nihon Kohden, Abbott Medical Japan, Fukuda Denshi, Boston Scientific Japan, Japan Lifeline, Nipro, and Biotronik Japan. K.T. has received honoraria from Bayer Yakuhin, Daiichi‐Sankyo, Kowa, MSD, Sanofi, and Takeda Pharmaceutical; and grants from Astellas Pharma, Abbott Vascular Japan, Bayer Yakuhin, Boehringer Ingelheim Japan, Boston Scientific Japan, Bristol‐Myers, Chugai Pharmaceutical, Daiichi‐Sankyo, Goodman, Japan Lifeline, Medtronic Japan, Mitsubishi Tanabe Pharma, MSD, Novartis Pharma, Otsuka Pharmaceutical, Sanofi, Takeda Pharmaceutical, and Terumo. All remaining authors declare no conflicts of interest.

## Data Availability

The data that support the findings of this study are available from the corresponding author upon reasonable request.
